# Supplementary material for: Incidence of Dysphagia and Comorbidities in Patients with Cervical Dystonia, Analyzed by Botulinum Neurotoxin Treatment Exposure
Source: Toxins (Basel). 2025 Mar 19;17(3):148. doi: 10.3390/toxins17030148 (PMC11945862; doi:10.3390/toxins17030148)
Supplement: Supplementary file 1 [file toxins-17-00148-s001.zip › toxins-3479896-supplementary.pdf]

Supplementary Materials: Incidence of Dysphagia and Comorbidities in Patients with Cervical Dystonia, Analyzed by Botulinum Neurotoxin Treatment Exposure

Richard L. Barbano[1], **Bahman** Jabbari[2], **Marjan** Sadeghi[3,*], **Ahunna** Ukah[3], **Emma** Yue[3], **Kimberly** Becker Ifantides[3], **Nuo-Yu** Huang[3] and **David** Swope[4]

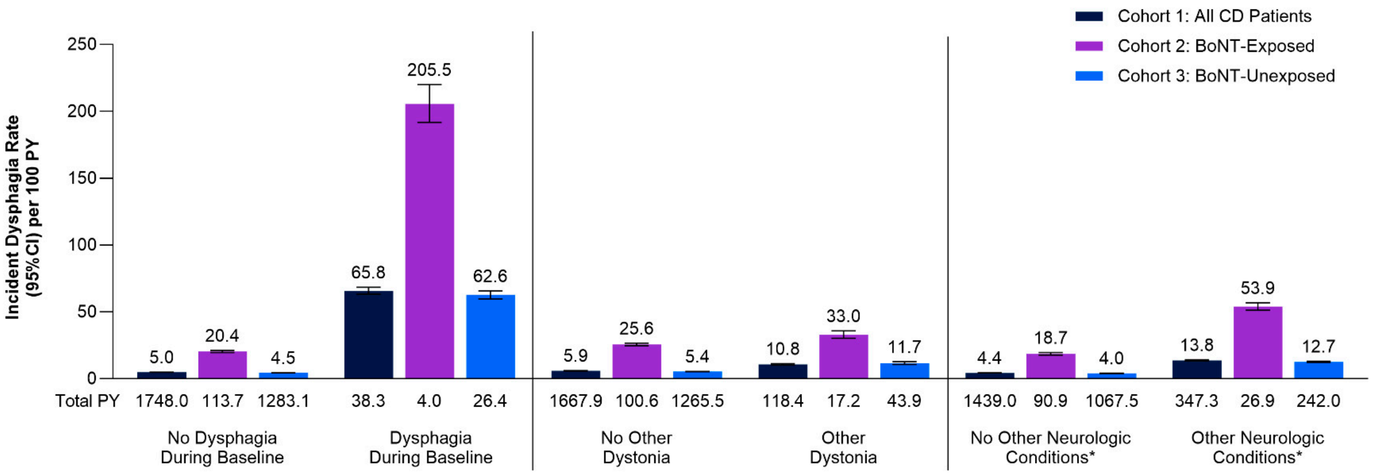

Figure S1. Incidence rates of dysphagia per 100 person-years in patients with dysphagia and presence or absence of baseline comorbidities by cohort. *Neurologic conditions that are known risk factors for dysphagia include stroke, Parkinson's disease, multiple sclerosis, Huntington's disease, Wilson's disease, gastroesophageal reflux disease, and other neuromuscular disorders. BoNT, botulinum neurotoxin; CD, cervical dystonia; CI, confidence interval; PY, person-years.

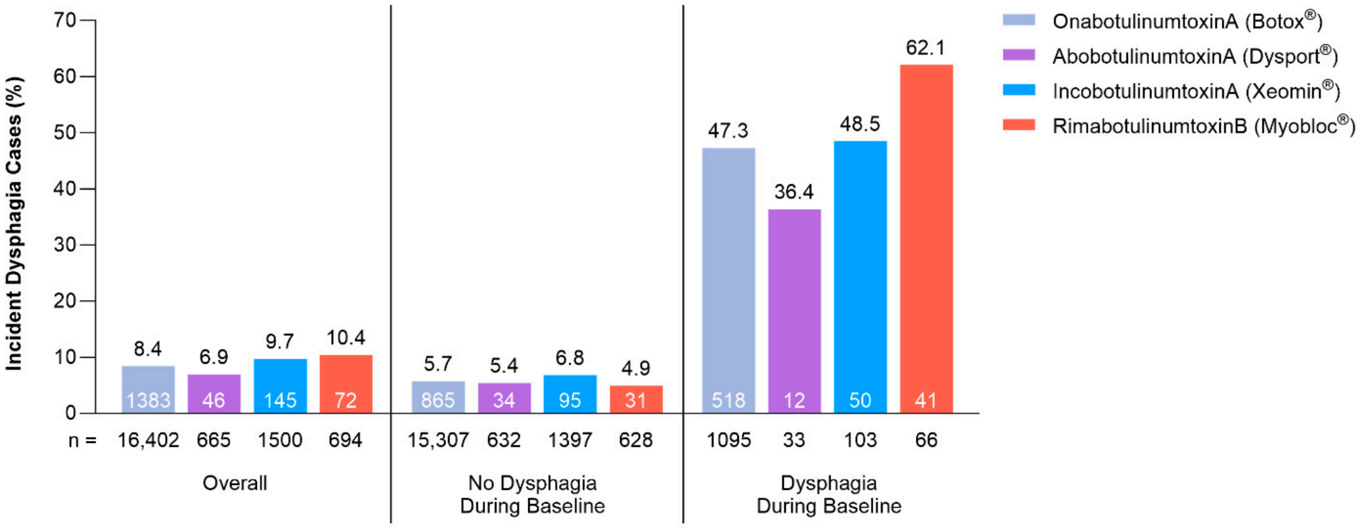

Figure S2. Proportion of patients with dysphagia by presence or absence of baseline dysphagia and BoNT product prescribed. Values within data bars (white text) indicate actual number of total cases of dysphagia.

Table S1. Initial patient identification and attrition.

Inclusion Criteria	Patients Remaining, n (%)	Patients Excluded, n (%)
Have at least two ICD-10 diagnoses for CD separated by at least 30 days from 2017/01/01 – 2021/9/30. The first CD diagnosis date is the CED	144,589	–
Number of patients from Claims alone	104,999	–
Number of patients from EHR alone	21,776	–
Number of patients from both Claims and EHR	17,814	–
≥180 days of continuous health plan enrollment (45-day gap allowed) prior to the CED	95,080 (65.8%)	49,509 (34.2%)
Patients ≥18 years of age	81,884 (86.1%)	13,196 (13.9%)
Cohort 1 eligible patients	81,884 (86.1%)	–
Exclude patients who had any BoNT injection in the neck area within 12 weeks prior to CED	80,398 (98.2%)	1486 (1.8%)
Cohort 2 eligible patients[a]	19,244 (23.9%)	–
Cohort 3 eligible patients[b]	61,154 (76.1%)	–

[a]≥1 BoNT injection in the neck are on or after CED; the index date was the first BoNT injection date. [b]No BoNT injection in the neck area on or after CED; the index date was the first CD diagnosis date. BoNT, botulinum neurotoxin; CD, cervical dystonia; CED, cohort entry date; EHR, electronic health records.

Table S2. Cervical dystonia ICD-10-CM diagnosis codes.

	Code
Cervical Dystonia	G24.1x - Genetic torsion dystonia<br>G24.3x - Spasmodic torticollis<br>M43.6x - Torticollis
Other Dystonia	G24.0x - Drug induced dystonia<br>G24.2x - Idiopathic nonfamilial dystonia<br>G24.4x - Idiopathic orofacial dystonia<br>G24.8x - Other dystonia<br>G24.9x - Dystonia, unspecified

ICD-10-CM, International Classification of Diseases, Tenth Revision, Clinical Modification.

Table S3. Dysphagia ICD-10-CM diagnosis codes.

	Code
Dysphagia	R13.1x<br>R13.10x -Dysphagia, unspecified<br>R13.11x -Dysphagia, oral phase<br>R13.12x -Dysphagia, oropharyngeal phase<br>R13.13x -Dysphagia, pharyngeal phase<br>R13.14x -Dysphagia, pharyngoesophageal phase<br>R13.19x -Other dysphagia

ICD-10-CM, International Classification of Diseases, Tenth Revision, Clinical Modification.
